# The Zebrafish as a Tool to Study Hematopoiesis, Human Blood Diseases, and Immune Function

**DOI:** 10.1155/2012/425345

**Published:** 2012-10-03

**Authors:** Jason Berman, Elspeth Payne, Christopher Hall

**Affiliations:** ^1^Departments of Pediatrics, Microbiology and Immunology and Pathology, Dalhousie University and IWK Health Centre, Halifax, NS, Canada B3K 6R8; ^2^Department of Haematology, University College London Cancer Centre, London, UK; ^3^Department of Molecular Medicine and Pathology, The University of Auckland, Auckland, New Zealand

Over the last decade, the zebrafish has cemented itself as a unique model system for providing new insights into the regulatory factors required for vertebrate hematopoiesis. In particular, the ease of genetic manipulation together with the transparency of embryos facilitating high resolution imaging has enabled the fate mapping of a host of blood cell lineages. Most notably, this has included the detailed evaluation of the origin and emergence of hematopoietic stem cells. Genetic conservation between zebrafish and mammals and the construction of well-annotated detailed genomic databases have permitted the use of a number of forward and reverse genetic approaches to study a variety of benign and malignant human blood disorders in this organism. These studies have revealed new molecular players underlying human phenotypes as well as providing platforms both for genetic screens to identify novel interacting partners as well as chemical modifier screens to reveal compounds that may represent new therapeutic strategies. Conserved hematopoietic cell biology extends across the innate and adaptive immune systems, fueling a recent growth of research focused on exploiting the advantages of the zebrafish system to examine vertebrate host-pathogen interactions and the contributions of individual cell subtypes to innate and adaptive immune responses.

This special issue highlights some of the most recent and profound contributions provided by the zebrafish model system to understand hematopoiesis, hematopoietic malignancies, and the vertebrate immune system. As a volume, it highlights the tremendous accomplishments achieved in these diverse areas of hematology using the zebrafish model to date and sets the stage for continued advancement in all spheres of hematology, oncology, and immunology using this highly genetically conserved, easily manipulated, and clearly visualized remarkable organism. 

In the paper entitled *“Novel insights into the genetic controls of primitive and definitive hematopoiesis from zebrafish models*,*”* R. Sood and P. Liu review the anatomic sites and developmental waves of primitive and definitive hematopoiesis and emphasize the conservation of critical transcription factors and other genes that regulate these processes. They highlight some of their own recent work in this field in which they utilize a zebrafish *runx1* mutant to identify novel insights into the role of *runx1* in definitive hematopoiesis and identify a hypomorphic allele of *gata1* that provides the opportunity to more precisely attribute the contribution of this transcription factor to various stages of erythroid development. 

In the report entitled *“Myelopoiesis and myeloid leukaemogenesis in the zebrafish,”* A. M. Forrester et al. highlight the conservation of myeloid gene regulation in zebrafish and describe the recent advances in this field. A number of studies and approaches are reviewed that have shed light on vertebrate neutrophil, monocyte, eosinophil, and mast cell development and provide a suite of *in vivo* tools to examine the perturbations associated with premalignant and malignant myeloid disease. 

Myeloid cells are key players in the innate immune system, an area of increasing investigation using the zebrafish model. A. H. Meijer et al. provide an overview of this area in their paper entitled *“Pathogen recognition and activation of the innate immune response in zebrafish*.*”* Conservation of toll-like receptors, nucleotide-binding oligomerization domain (NOD)-like receptors, and other key members of the innate immune response are discussed and examined in the context of a number of bacterial pathogens. Novel immune-type receptors (NITRs) and functional orthologues in zebrafish of mammalian NK cell receptors are characterized in the paper by J. Yoder's group entitled *“Development and characterization of anti-Nitr9 antibodies*.*”* C. Wittmann et al. outline the critical role of hydrogen peroxide as a mediator of inflammatory responses in the zebrafish in their paper entitled *“Hydrogen peroxide in inflammation:messenger, guide, and assassin*.*”* Neutrophil behaviour in response to wounds is dissected in more detail in two papers entitled *“Neutrophil reverse migration becomes transparent with zebrafish”* by T. W Starnes and A. Huttenlocher's group and *“Drift-diffusion analysis of neutrophil migration during inflammation resolution in a zebrafish model”* by S. A. Renshaw et al. Huttenlocher's group takes advantage of a neutrophil-specific Lyn oxidation mutant to demonstrate that this Src family kinase is a critical link between hydrogen peroxide produced at the site of a wound and neutrophil chemoattraction. The imaging capabilities of the zebrafish and photoconversion techniques are subsequently exploited by both groups to reveal the process of neutrophil reverse migration for the first time. The purpose of this phenomenon and ultimate fate of these reversely travelling cells remain to be determined. However, the zebrafish is likely to serve a key role in further elucidating the factors underlying this process.

Platelet development and hemostasis in the zebrafish is next addressed in two papers entitled *“Zebrafish thrombocytes: functions and origins”* by P. Jagadeeswaran et al. and *“Characterization of zebrafish von Willebrand factor reveals conservation of domain structure, multimerization, and intracellular storage”* by J. A. Shavit et al. These reports set the stage for the zebrafish to provide new insights into platelet biology and model human bleeding disorders.

This special issue also includes a number of papers highlighting the utility of the zebrafish as a tool in dissecting oncogenic pathways in leukemia pathogenesis, identifying novel therapies, and improving stem cell transplantation. F. E. Moore and D. M. Langenau summarize the transgenic models of leukemia that have been developed by their laboratory and others in their paper entitled *“Through the looking glass: visualizing leukemia growth, migration, and engraftment using fluorescent transgenic zebrafish.”* They present the opportunities provided by the transparency of zebrafish embryos and fluorescent labeling to study leukemia cell engraftment, homing, and frequency of leukemia propagating cells. These transgenic leukemia models provide a platform both for further genetic interrogation and high throughput drug screening. In their paper entitled *“Genomic amplification of an endogenous retrovirus in zebrafish T-cell malignancies,”* J. K. Frazer et al. utilize array comparative genomic hybridization (aCGH) on the genomes of three zebrafish T-cell leukemia transgenic lines to identify a novel oncogenic retrovirus. Y. Zhang and J. R. Joanna Yeh describe the process for conducting chemical screens in zebrafish embryos in their paper entitled *“In vivo chemical screening for modulators of hematopoiesis and hematological disease”* and highlight the tremendous advantages and opportunities inherent in this approach. In particular, they describe the identification of the prostaglandin pathway and COX proteins in two separate screens: as positive regulators of hematopoietic stem cell development and as targets for inhibition in AML1-ETO driven myeloid disease. Finally, J. L. O. de Jong and L. I. O. Zon have contributed, *“Histocompatibility and hematopoietic transplantation in the zebrafish,”* whereby they extend the zebrafish model to studies of matched allogeneic stem cell transplantation, with potential to quantify engraftment and model graft versus host disease.

*Jason Berman* *Elspeth Payne* *Christopher Hall*

